# Birds and people in semiarid northeastern Brazil: symbolic and medicinal relationships

**DOI:** 10.1186/1746-4269-9-3

**Published:** 2013-01-08

**Authors:** Dandara Monalisa Mariz Bezerra, Helder Farias Pereira de Araujo, Ângelo Giuseppe Chaves Alves, Rômulo Romeu Nóbrega Alves

**Affiliations:** 1 Departamento de Sistemática e Ecologia, Universidade Federal da Paraíba, 58051-900, Campus I, Brasil; 2 Departamento de Ciências Biológicas, Universidade Federal da Paraíba, 58051-900, Campus I, Brasil; 3 Departamento de Biologia, Universidade Federal Rural de Pernambuco, Rua Manoel de Medeiros, S/N, Dois Irmãos, 52171-900, Recife, Pernambuco; 4 Departamento de Ciências Biológicas e da Saúda, Universidade Estadual da Paraíba, 58109-753, Paraíba, Brasil

**Keywords:** Caatinga, Beliefs, Ethno-ornithology, Ethnozoology, Zootherapy, Palavras-chave, Caatinga, Crenças, Etnoornitologia, Etnozoologia, Zooterapia

## Abstract

**Abstract:**

**Background**

At least 511 species of birds occur in the semiarid region of northeastern Brazil and many of them interact with human populations in a number of different ways, including their use in zootherapeutics and their links with local beliefs.

**Objective**

The present work examined these types of birds/human interactions (use in zootherapeutics and their links with local beliefs) in the semiarid region of Rio Grande do Norte State in northeastern Brazil.

**Methods**

Information was obtained through semi-structured interviews with 120 local residents.

**Results**

A total of 16 wild bird species distributed among 11 families were found to interact directly with humans, with Columbidae being the best represented family. Seven species were identified in the medicinal category, five were related to symbolic aspects, while four species were identified as being related to both categories (medicinal and symbolic).

**Conclusion**

The accumulated folk knowledge, beliefs, and practices involving the avifauna in the semiarid region of Brazil, whether symbolic or medicinal, demonstrated the cultural importance of this vertebrate group to local human populations and revealed a belief system intrinsically related to cynegetic practices in the region.

**Portuguese abstract:**

**Introdução**

No semiárido do Brasil ocorrem 511 espécies de aves, algumas das quais interagem frequentemente com as populações humanas locais de diferentes formas, incluindo o uso como zooterápico e a inserção em crenças.

**Objetivo**

Esta pesquisa registrou as aves diretamente associadas a essas formas de interação (o uso como zooterápico e a inserção em crenças) na região semiárida do Estado do Rio Grande do Norte, Nordeste do Brasil.

**Métodos**

As informações foram obtidas através de entrevistas semiestruturadas aplicadas a 120 moradores locais.

**Resultados**

Foram registradas 16 espécies de aves silvestres distribuídas em 11 famílias, sendo Columbidae a que apresentou o maior número de representantes. Sete espécies foram indicadas apenas na categoria medicinal, cinco espécies apenas relacionadas a aspectos simbólicos e quatro foram indicadas como relacionadas a ambas as categorias (medicinal e simbólica).

**Conclusão**

Os conhecimentos, crenças e práticas relacionadas à avifauna no semiárido brasileiro, seja no aspecto simbólico ou medicinal, demonstra a importância cultural que esse grupo de vertebrados representa para as populações locais, revelando um sistema de crenças que está intrinsicamente relacionado às práticas cinegéticas na região.

## Background

Brazil has one of the most diversified avifaunal populations in the world, with more 1,832 known species [1]. At least 511 avian species have been recorded in the semiarid region (which is dominated by steppe-savannah vegetation that is locally denominated “caatinga”) [2,3], and a number of these birds are used by local human populations [4-14].

Ancient relationships have been established between birds and human populations, and these animals are present in the day-to-day actions and thoughts of human cultures in many ways [15,16]. Birds are usually considered in terms of their roles as pets or as sources of food [8,9,11,14,17], although these animals have other important forms of interaction with humans in medicinal and symbolic spheres [5,15,18-25].

Among the many types of ethno-zoological connections that exist between humans and animals are symbolic-ritualistic relationships that, in the case of birds, refer to omens culturally associated with these animals [26] and with the use of their body parts in magical/religious rituals [22,24,25]. In regards to omens, these divinations may be related to meteorological phenomena [27,28] and/or have religious dimensions [22,29,30].

Birds are among the animals most frequently used in folk medicine in Brazil [6,7,19,23,31-40] and other countries of the world [20,41-49]. Zootherapeutic knowledge and practices are generally orally transmitted from generation to generation, and are associated with a wide array of cultural aspects [50]. Interrelationships between popular beliefs and zootherapy have been reported in many different localities in Brazil [6,9,31,51-54] as well as in other countries [48,49,55,56]. As such, the use of animal-derived substances should be viewed from a cultural perspective as these medicinal practices are organized into cultural systems [57] and form through the integration of belief systems (*kosmos*), bodies of accumulated knowledge (*corpus*), and productive practices (*práxis*) [58].

Both the hunting and capture of bird species have been shown to affect their natural populations – with immediate and evident ecological implications [8,9,14]. As such, studies are needed that can reveal the different forms of interactions between people and birds and investigate questions related to their ecology, economic uses, and symbolic functions that could aid future conservation efforts [59]. Most ethnozoological research on birds has focused on their use and commerce as pets or food, while studies examining the importance of these animals as components of belief systems have been largely neglected. These types of studies are very important from a conservation point of view, however, because human perceptions of the symbolic value of an animal can be associated with either protective or destructive attitudes towards them. Within this context, the present work surveyed local human populations of hunters and ex-hunters living in the semiarid region of northeastern Brazil to identify the bird species hunted and utilized as therapeutic resources and/or cited in belief systems in order to evaluate the implications of these relationships on the conservation of the local avifauna.

## Methodology

### Study area

**Figure 1:**
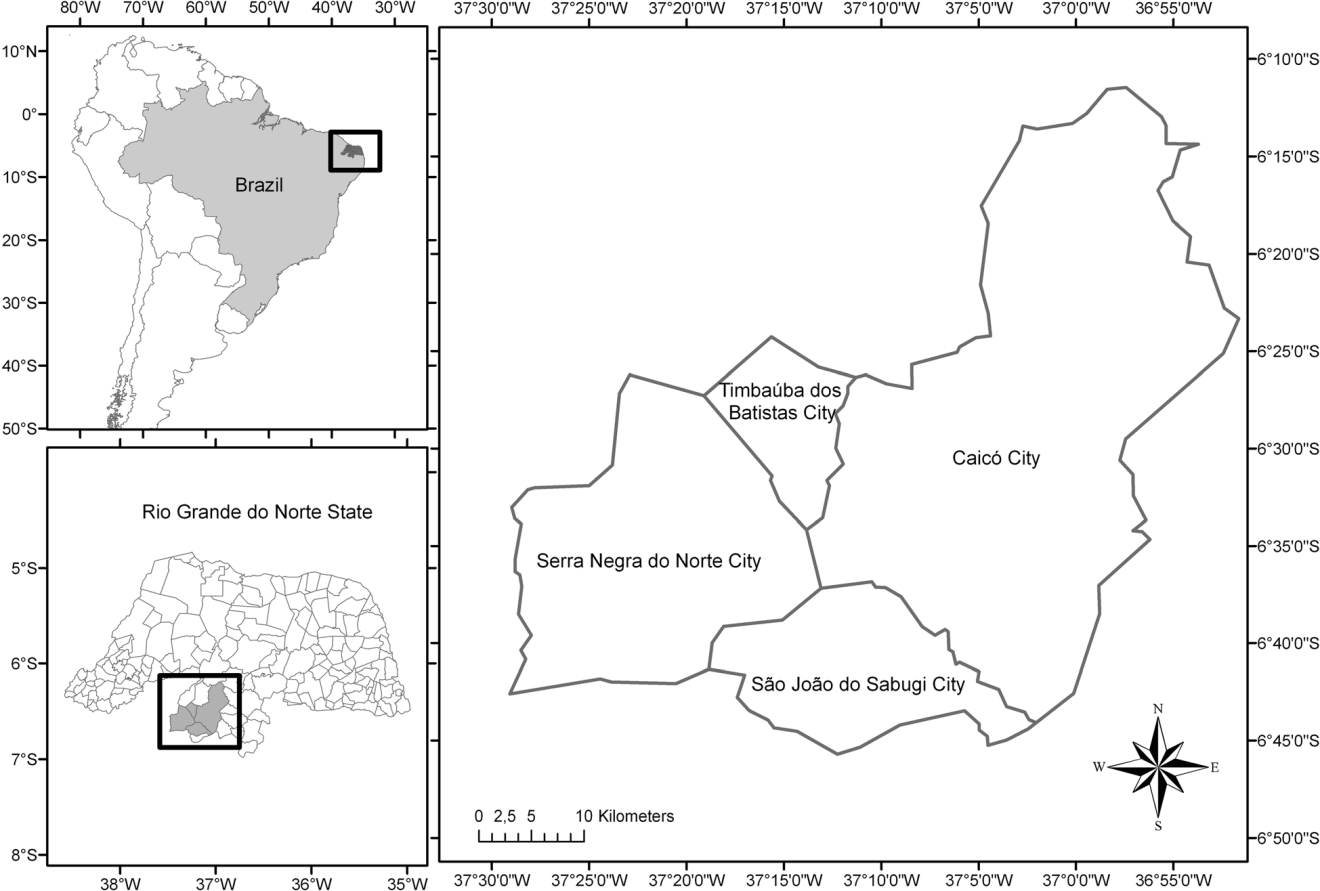
Map of the study area, indicating the municipalities of Caicó, São João do Sabugi, Serra Negra do Norte, and Timbaúba dos Batistas, in Rio Grande do Norte State, Brazil.

The study area included the municipalities of Caicó, São João do Sabugi, Serra Negra do Norte, and Timbaúba dos Batistas (Figure 1), located in the *Seridó* region of the state of Rio Grande do Norte in northeastern Brazil. The *Seridó* region occupies an area of 10,955 km^2^[60] (approximately 20.74% of the state), most of which is covered by *Caatinga* dryland vegetation that has been heavily impacted by anthropogenic use and subject to desertification [61].

The regional climate is dry and hot (classified as Bswh in the Köppen system) with an average annual temperature of 27.5°C [62]. The rains are concentrated in the austral Summer/Fall (between January and April). The local vegetation is classified as steppe-savannah, locally *caatinga* denominated, with a number of distinct characteristics that causes it to be considered "*Seridó caatinga*" [63,64].

### Characterization of the target population

The human populations in the municipalities studied are largely composed of people involved in small-scale agricultural activities such as subsistence agriculture, animal husbandry (goats, sheep, and cattle), and the service sector (e.g., small businessmen, teachers, etc.) who also undertake cynegetic activities in the region. The populations in the study area consider themselves to be “sertanejos”. In the categories developed by [65] to describe traditional populations, the sertanejos/vaqueiros correspond to traditional non-indigenous populations that occupy inland areas of northeast Brazil and can also advance into the semiarid *caatinga* region.

We interviewed a total of 120 people including hunters and ex-hunters living in the four municipalities cited above. Of these, 77.5% now live in urban areas, while the remaining 22.5% reside in rural areas. In spite of the fact that most of the interviewees lived in urban situations they still retained strong links with the rural zone, where they not only practiced their professions but also captured birds. The high percentage of people in urban areas actively using wild birds reflects their relatively recent migration from rural to urban areas as well as the transplanting of rural cultural practices to urban situations.

### Sampling procedures and data analysis

Fieldwork was undertaken during the period between September/2009 and March/2010. Information was gathered through the use of interviews held with local inhabitants who utilized or interacted with wild birds in some manner. With the aid of local community leaders we were able to identify key-informants in each municipality and the selection of interviewees then developed through the “snowball” technique [66], which consists of identifying additional informants based on recommendations of earlier interviewees.

Ethno-ornithological data was obtained through the use of semi-structured interviews [67] and direct observations [68]. The semi-structured interviews were directed towards identifying the types of birds that were captured, their forms of use and symbolic aspects associated. The interviews were undertaken individually, and most were digitally recorded; the interviews were subsequently transcribed and organized into a standardized data base. We used field notebook for notes, especially in interviews that could not be electronically recorded.

The birds cited by the interviewees were identified to the species level using published field guides [69,70], by direct sightings, and by photographic documentation during the interviews. The scientific nomenclature utilized in the present study follows the Brazilian Commission of Ornithological Records [1].

The general goals and objectives of the study were outlined to the potential informants in accessible language before beginning the interviews. This research project was approved by the Research Ethics Committee of the Lauro Wanderley University Hospital of the Federal University Paraíba (protocol CEP/HULW nº. 0008.0.126.000-10).

## Results and discussion

Of the 120 interviewees, only 33.3% mentioned the use of bird species in medicinal or symbolic functions, while the remainder cited their use as food sources or as pets. This low percentage may reflect a low level of use of wild birds for medicinal or symbolic practices, or it may be related to a decrease in the transmission of this type of knowledge to younger generations. Additionally, knowledge about some species was limited to a restricted number of interviewees (see Table 1), which corroborates with the hypothesis of a decrease in the rate of transmission of this type of knowledge in the regions studied.

**Table 1:** Birds cited by informants in the semiarid region of Rio Grande do Norte State in northeastern Brazil as having medicinal and/or symbolic attributes: identification, categories and subcategories, uses and purposes (M- medicinal; S-symbolic), and number of citations per species

**Families/species**	**Vernacular name**	**Category (subcategory)**	**Uses and purposes**	**Citations**
Tinamidae				
*Nothura boraquira* Spix, 1825	White-bellied Nothura	Medicinal (cures), Symbolic (protection)	**M**- Administering an infusion made from the feathers of this bird will supposedly cure someone bitten by a snake. A soup made from the cooked meat of this bird can cure colds. **S- **Carrying a feather from this bird will protect a person from disagreeable events.	4
Carthartidae				
*Coragyps atratus* Bechstein, 1793	Black Vulture	Medicinal (cures)	**M-** The liver of this bird is extracted and cooked and then reduced to a powder. If this powder is added to the drink or food of an alcoholic, without that person being aware of it, they will supposedly be cured of alcoholism. Ingesting fat from the bones of this vulture will cure aching bones.	5
Cariamidae				
*Cariama cristata* Linnaeus, 1766	Red-legged Seriema	Medicinal (prevention) Symbolic (protection)	**M**- Using a necklace made from the feathers of this bird will help avoid serious reactions to snake bites. **S**- Using a necklace made from the feathers of this bird will protect the hunter and his dog from snakebites.	2
Columbidae				
*Columbina minuta* Linnaeus, 1766	Plain-breasted Ground-Dove	Medicinal (cures)	**M**- Drying the foot this bird and eating it raw, or preparing an infusion with the feces of this animal, are believed to cure colds.	2
*Columbina talpacoti* Temminck, 1811	Ruddy Ground-Dove	Medicinal (cures)	**M-** Drying the foot this bird and eating it raw, or preparing an infusion with the feces of this animal, are believed to cure colds.	2
*Columbina squammata* Lesson, 1831	Scaled Dove	Medicinal (cures)	**M-** Drying the foot this bird and eating it raw, or preparing an infusion with the feces of this animal, are believed to cure colds.	2
*Columbina picui* Temminck, 1813	Picui Ground-Dove	Medicinal (cures) Symbolic (omens)	**M**- Drying the foot this bird and eating it raw, or preparing an infusion with the feces of this animal, are believed to cure colds. **S-** The call of this bird is believed to predict disagreeable events.	3
*Patagioenas picazuro* Temminck, 1813	Picazuro Pigeon	Symbolic (omens)	**S**- It is believed that this bird attracts disagreeable events.	1
*Leptotila verreauxi* Bonaparte, 1855	White-tipped Dove	Medicinal (cures)	**M**- Eating the cooked meat of this bird will cure morning sickness during pregnancy.	5
Psittacidae				
*Aratinga cactorum* Kuhl, 1820	Cactus Parakeet	Medicinal (cures)	**M**- Consuming a brew made from the meat of this bird is believed to facilitate the eruption of new teeth in children.	1
Trochilidae				
*Chlorostibon lucidus* Shaw, 1812	Glittering-bellied Emerald	Symbolic	**S-** Eating the heart of this bird will make a hunter more successful.	1
Corvidae				
*Cyanocorax cyanopogon* Wied, 1821	White-naped Jay	Medicinal (cures), Symbolic (protection)	**M**- If this bird is raised as a pet and fed with leftover food of a person afflicted with asthma or shortness of breath it will cure this illness. Applying an infusion made with the feathers of this bird will supposedly cure Chagas disease. **S**- A bird kept as a pet in the house can help prevent disagreeable events, as it is believed to be capable of foretelling them.	25
Turdidae				
*Turdus rufiventris* Vieillot, 1818	Rufous-bellied Thrush	Symbolic (climate)	**S**- The song of this bird is believed to foretell rainfall.	1
Mimidae				
*Mimus saturninus* Lichtenstein, 1823	Chalk-browed Mockingbird	Medicinal (cures)	**M**- Eating the cooked meat of this bird will cure morning sickness during pregnancy.	1
Icteridae				
*Icterus jamacaii* Gmelin, 1788	Campo Troupial	Symbolic (omens)	**S**- Keeping this bird as a pet can attract disagreeable events.	1
Fringillidae				
*Euphonia chlorotica* Linnaeus, 1766	Purple-throated Euphonia	Symbolic (omens)	**S**- The song of this bird is believed to attract disagreeable events.	2

**Figure 2:**
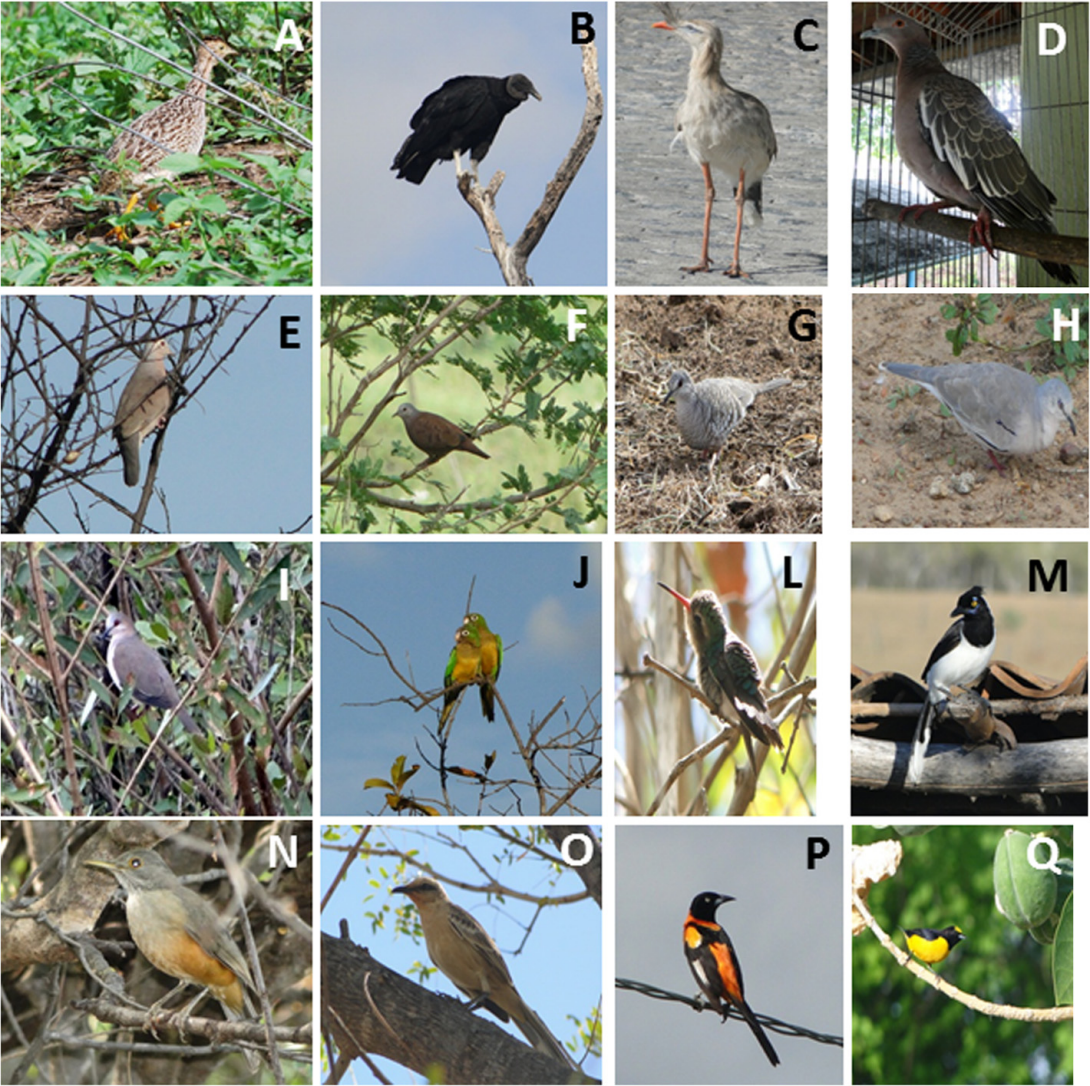
**Bird species that were cited in popular medicine, or associated with local belief systems by interviewee in the state of Rio Grande do Norte: A- *Nothura boraquira*, B- *Coragyps atratus*, C- *Cariama cristata*, D- *Patagioenas picazuro*, E- *Columbina minuta*, F- *Columbina talpacoti*, G- *Columbina squammata*, H- *Columbina picui*, I- *Leptotila verreauxi*, J- *Aratinga cactorum*, L- *Chlorostilbon lucidus*, M- *Cyanocorax cyanopogon*, N- *Turdus rufiventris*, O- *Mimus saturninus*, P- *Icterus jamacaii*, Q- *Euphonia chlorotica.*** Photos: A. John Medcraft (2009); B-Q. Dandara Bezerra (2010, 2011).

Sixteen bird species were identified that were used in popular medicine or associated with local belief systems (Figure 2). These species belonged to 11 families, with Columbidae having the highest number of species mentioned (37.5%) (Table 1). These species are not cited in the Brazilian list of threatened birds [71], and only in the “Least Concern” category prepared by the International Union for the Conservation of Nature [72]. There is currently no information available in the literature about the degree of conservation of the species present in the study region, and a list of locally threatened species would provide important information about cynegetic pressure on regional native birds.

The categories and subcategories used in this work are classified according to the perspective of Western science, as the local populations do not recognize any sharp distinctions between natural, supernatural, magical, religious, or medicinal spheres [31,58]. Using the typology proposed by Marques [18,73], the present research considered the following categories and subcategories: a) medicinal: birds used in curative treatments (subcategory cures) or in preventative measures (subcategory prevention); b) symbolic: birds that humans consider capable of divining events related to the climate and the weather (subcategory climate), events that announce some misfortune (subcategory omens), or birds that are used in attempts to avoid certain problems or bring good fortune to cynegetic activities (subcategory protection).

According to the categories and subcategories proposed, 11 bird species were placed in the medicinal category (*Nothura boraquira, Coragyps atratus, Cariama cristatus, Columbina minuta, Columbina talpacoti, Columbina squammata, Columbina picui, Leptotila verreauxi, Aratinga cactorum, Cyanocorax cyanopogon,* and *Mimus saturninus*), and of these species, 10 were included in the subcategory cures, while only one (*Cariama cristatus*) was included in the subcategory prevention. Nine species (*N. boraquira, C. cristatus, C. picui, Patagioenas picazuro, Chlorostibon lucides, C. cyanopogon, Turdus rufiventris, Icterus jamacaii,* and *Euphonia chlorotica*) were included in the symbolic category, with four (*N. boraquira, C. cristatus, C. lucides,* and *C. cyanopogon*) being included in the subcategory protection, one (*T. rufiventris*) in the subcategory climate and four (*C. picui, P. picazuro, I. jamacaii,* and *E. chlorotica*) in the subcategory omens (see Table 1).

Seven bird species (*Coragyps atratus, C. minuta, C. talpacoti, C. squammata, L. verreauxi, Aratinga cactorum,* and *Mimus saturninus*) were included only in the medicinal category, five species (*P. picazuro, C. lucides, T. rufiventris, I. jamacaii,* and *E. chlorotica*) were related only to symbolic aspects, while four species (*N. boraquira, C. cristatus, C. picui,* and *C. cyanopogon*) were identified as belonging to both categories (medicinal and symbolic). It was noted that the medicinal and symbolic qualities attributed to the bird species cited by the hunters and former hunters were related in a general way with a system of folk beliefs transmitted down through generations.

### Birds as zootherapeutic resources

Eleven bird species were cited by the interviewees as therapeutic resources in the municipalities surveyed (see Table 1). Five species had already been reported in the literature, but six were new records of medicinal birds for Brazil (*Aratinga cactorum, Columbina minuta, C. talpacoti, C. picui, C. squammata,* and *Mimus saturninus*). Summing these new species to the 47 medicinal bird species already recorded in the literature [35,57] indicates that at least 53 bird species are used in folk medicinal practices in Brazil. The uses of some of these species, such as *Gallus gallus*, *Coragyps atratus*, *Nothura boraquira*, and *Cyanocorax cyanopogon* are disseminated in many parts of Brazil [5-7,21,31-34,36,38,39,74-76]. New research initiatives concerning medicinal animals will be needed in various regions of Brazil as the true number of medicinal species must certainly exceed that presently known (see [35]). Cataloging species and their zootherapeutic uses is important because of the cultural values they represent and because this information could also be useful in terms of conserving these species. It must be emphasized, however, that there is no information available in the literature concerning the pharmacological efficiency of the therapies cited in the present study. Very few studies, in fact, have been undertaken to investigate the efficiency of any of the animals used in traditional medicines [77-79] although a limited number of workers have examined this subject e.g. [80-82]. As such, inventories of fauna-derived medicines should be subsidized with pharmacological studies that could validate (or not) the medicinal properties of animals used in folk medicine [37,79].

Medicinal uses of animals are widely disseminated throughout the world. Birds are the second most frequently used vertebrates for medicinal purposes in India [46], and a similar survey undertaken in public markets in Nigeria recorded 199 bird species used in traditional medicine [83]. Kizung *et al.*[41] studied the uses of birds by human populations in the Democratic Republic of Congo and reported that 11.7% of the 76 species cited by the informants were used in traditional medicinal practices. Williams *et al.*[84] reported that at least 354 species of birds are used for tradicional medicine in 25 African countries.

The use of birds as therapeutic resources in the present study area is based on folk knowledge that was transmitted by older members of the population. This situation follows a tendency previously noted by various authors who pointed out that zootherapeutic knowledge about birds is, as a general rule, passed down orally by older members of the community – indicating the cultural value of this accumulated knowledge [5,7,21,32-34,36].

The White-naped Jay (*Cyanocorax cyanopogon*) had the greatest number of citations among the birds attributed with having zootherapeutic qualities in all of the municipalities studied here. The principal medicinal role of this bird, according to the informants, involves the use of live specimens to treat asthmatic problems. The interviewees reported that this malady is transferred to the bird when it is fed with the leftovers from the plate of an ailing person. This same prescription was also described in the semiarid regions of the states of Pernambuco, Paraíba, and Ceará, all of which are located in northeastern Brazil [5,6,14]. Beliefs associated with the “transfer of illnesses” to animals or plants that are placed close to a sick person or to an infected area have been reported by a number of other authors [5,6,14,36,48]. Ribeiro et al. [74], for example, noted that the simple act of sitting on a sloth skin (*Bradypus spp.*) was used by local populations in southern Bahia State to treat back problems. Bernitez [48] reported that interviewees from the province of Granada in Spain recommend placing a snakeskin close to the head of a patient to prevent (or treat) headaches.

In relation to the Red-legged Seriema (*Cariama cristata*), the informants indicated that its feathers are used as amulets to protect hunters and their dogs from snake bites. This is in agreement with the findings of Alves & Rosa [32] and Souto *et al.*[40], who reported use of animal sub-products for making amulets to prevent or treat illnesses affecting humans and their domestic animals. The manners in which the White-naped Jay and the Red-legged Seriema are used reaffirms the observation that popular zootherapeutic treatments are part of a complex medical system that incorporates, among other popular health practices, rituals and magical prophylaxes such as amulets, talismans, and transference [85].

Other species cited by the interviewees included the Black Vulture (*Coragyps atratus*), which has widely disseminated medicinal uses in various localities in Brazil (especially in the northeastern region). This bird is popularly prescribed to treat asthma and/or alcoholism [6,15,31,33,38,74,75], but was only prescribed in the study area for treating alcoholism – the same indication previously recorded by researchers in the states of Paraíba and Pernambuco [5,7]. According to the interviewees, the liver of the vulture must be extracted and roasted and then ground into a powder that is placed in the drink or food of an alcoholic – but without that person being aware of it. This type of prescription, in which the administration of a medicine derived from an animal part would only be effective if the patient did not know it was being used, has been reported by other workers [5,7,35,48,74,75,86-88].

It is important to note that many zootherapeutic products are derived from birds captured for other purposes by the hunters who were interviewed. *Cariama cristata* (Red-legged Seriema), *Nothura boraquira* (White-bellied Nothura), *Leptotilila varreauxi* (White-tipped Dove), and the genus *Columbina* (Ground-Dove), for example, are hunted primarily for cynegetic purposes – but also furnish products with therapeutic uses. The use of these animal parts can be viewed as maximizing resource benefits from the rather limited local ecosystems [89].

### Birds associated with symbolic aspects

Nine bird species in the research area were found to be associated with beliefs or superstitions (see Table 1): *Euphonia chlorotica* (Purple-throated Euphonia)*, Icterus jamacaii* (Campo Troupial)*, Cyanocorax cyanopogon* (White-naped Jay)*, Nothura boraquira* (White-bellied Nothura)*, Cariam cristata* (Red-legged Seriema) the hummingbird *Chlorostibon lucidus* (Glittering-bellied Emerald), *Columbina picui* (Picui Ground-Dove), *Patagioenas picazuro* (Picazuro Pigeon), and *Turdus rufiventris* (Rufous-bellied Thrush). According to the interviewees, knowledge concerning these beliefs is acquired from older people or shared among friends during cynegetic activities. Although the interviewees might express certain doubts about these folk beliefs, they are considered traditional cultural elements in the region and the informants felt that it was preferable to respect them. Marques [90] observed that beliefs constitute important cultural mechanisms that serve to impose limits on the conduct and practices of community members, making certain ecological interactions socially acceptable or not.

The interviewees cited birds associated with various symbolic subcategories. These subcategories included attributes that varied from birds that supposedly bring good fortune to those that capture them, those that are considered bad omens, to those that can predict events related to the climate and the weather.

Bird vocalizations are often considered presages of natural or supernatural occurrences (ornithological divinations) and are classified according to the predicted events. The beliefs associated with the species *Euphonia chlorotica* (Purple-throated Euphonia) and *Columbina picui* (Picui Ground-Dove) in the study area were associated with the subcategory omens (birds whose calls are attributed with the power of predicting disagreeable events). A similar perception was documented among inhabitants of the Genipabu Environmental Protection Area in Rio Grande do Norte State, where Torres *et al.*[91] reported that the hummingbird *Eupetomena macroura* is considered to be associated with negative events. Costa-Neto [18] noted a superstition associated with the owl *Tyto alba*, with its calling being considered a bad omen.

In some cases, the informants indicated that they believed that certain birds if kept as pets could aid in avoiding disagreeable events because they are capable of predicting such occurrences, such as the White-naped Jay (*Cyanocorax cyanopogon*), which is included in the subcategory protection. In the case of this specific species, this belief may be explained in part by its characteristic ecological behavior, which according to Major et al. [92] consists of warning of the approach of any type of predator by its loud call. One informant reported that this bird would raise an alert if a snake appeared. As such, its calling serves as a type of alarm that could aid in preventing misfortune. A similar example was described by Marques [29], who reported a taboo against hunting specimens of *Vanellus chilensis*, a possible response to the territorial behavior of this species that uses its strident vocalizations to warn against the approximation of animals (human or otherwise).

*Icterus jamacaii* (Campo Troupial) and *Patagioenas picazuro* (Picazuro Pigeon) were considered by the interviewees consulted in this survey to be animals that can attract disagreeable events if kept as pets. According to Colding and Folke [93], taboos can be defined as unwritten social rules that regulate human behavior – informal institutions that can limit and define the use of ecosystem resources in certain contexts and thus take on conservation roles. In the cases of the species cited here, however, these beliefs were not found to be associated with any decrease in the capture rates of these animals, as the interviewees indicated that they continued to be hunted. *Pagioenas picazuro* is highly valued as a food source and *Icterus jamacaii* is commonly cited as a pet. These apparent contradictions between beliefs and usages reflect the life experiences of each individual, for as Toledo and Barrera-Bassols [94] observed, each person has his/her own *kosmo-corpus-praxis* complex (beliefs-knowledge-practices) that tune their experiences in response to three information sources: historically associated experiences, socially shared experiences, and individual experiences. On the other hand, the negative characteristics associated with the Purple-throated Euphonia (*Euphonia chlorotica*) may function to protect this species – and only five of the interviewees reported that they captured or raised these birds. This information may be useful in generating testable hypotheses related to ethno-conservation. A similar example was cited by Marques [29] and Farias *et al.*[30] that consisted of a total taboo protection of *Fluvicola negenta* (Masked Water-tyrant) encountered in northeastern Brazil. According to popular beliefs, this species helped wash the clothes of the infant Jesus (or his mother, Our Lady, in the Catholic tradition), so that killing or capturing it was effectively prohibited by popular Catholicism.

Still within the symbolic perspective, *Turdus rufiventris* (Rufous-bellied Thrush) is associated with the climate subcategory (in which bird vocalizations are believed to have the power of presaging the weather and climatic events). The calls of this bird were also cited by Marques [27] and Araujo *et al.*[28] (who documented various bird species considered presages of rainfall in the dry northeastern region of Brazil) as being considered to predict rainfall. Marques [29] noted that birds occupy a privileged position among the faunal elements incorporated into popular Catholic religious beliefs – and in many cases this consideration is intimately related to their vocalizations. Other workers have likewise noted cultural relationships associated with bird vocalizations [18,26,28,95,96].

Beliefs associated with the species *Nothura boraquira* (White-bellied Nothura), *Cariama cristata* (Red-legged Seriema), and *Chlorostibon lucidus* (Glittering-bellied Emerald) in the research area were not related to their songs but rather to uses of their body parts, which were used to aid hunters by protecting them against poisonous snakes and by increasing their luck. These birds appear to have significant cultural importance to hunters, as the first two were cited within medicinal and symbolic contexts related to cures, prevention, and protection during hunting activities. Similarly, other workers have reported the use of bird body parts as amulets or talismans, including Costa-Neto [18] who cited use of the feathers of the Ferruginous Pygmy-owl (*Glaucidium brasilianum*) by local populations in the Amazon basin to make amulets to attract good health as well as luck in games of chance and in love. In their study of public markets in Boa Vista in northern Brazil, Pinto & Maduro [53] recorded the popular use of body fat from Crimson-hooded Manakin (*Pipra aureola*) to make perfumes used to attract sexual partners. Already in study of public markets in Belém e Teresina in northern and northeast Brazil, respectively, Alves et al. [25] recorded the popular use of whole animal from Band-tailed Manakin (*P. fasciicauda*) to attract business; good luck/money; and perform and umbanda rituals.

In addition to the classification system adopted here, which differentiates between medicinal interactions and those of a symbolic nature, there are many close associations between these two forms of interaction. The fact that birds have a place of importance in popular medicine can be considered an important aspect of the worldview of the people directly involved in their use. Examples of the explicit intersection between symbolism and zootherapy include the belief that a certain behavior of the White-naped Jay bird (*Cyanocorax cyanopogon*) – feeding on the leftovers from the plate of a sick person – can help cure that patient, the belief that the simple presence of an animal (or part of an animal, as in the case of the feathers of *Cariama cristata*) can protect humans and domestic animals from attacks by predators, and the belief that to obtain a cure the patient must not be aware of detailed information about the animal products he/she is ingesting (as in the necessity of hiding the powdered liver of the vulture *Coragyps atratus* in treating alcoholism).

As was noted in our study, birds have an enormous cultural value and are extensively used in multiple practices with various medicinal and spiritual functions. Potential conservation links with these types of interactions should be more closely examined as they can vary from species to species, and these studies must take into account factors such as habitat predation and capture for other non-medicinal purposes – which are evident causes of at least part of the population declines noted for many species [57,97]. It is also important to emphasize that most of the zootherapeutic products cited here employ animal subproducts that would rarely used for any other purpose. Examples of this are *Nothura boraquira* and *Cariama cristata* and various species of the genus *Columbina*; these birds are hunted for food, but their feathers also serve as medicinal and symbolic subproducts (the former two species), while the feet of a number of species of the genus *Columbina* are used for zootherapeutic purposes. *Cyanocorax cyanopogon* demonstrates overlapping in the study area uses as a bird with important medicinal and symbolic attributes that is also appreciated as a pet. As such, the true motive for the cynegetic pressure on a given animal species may not be directly related to any single use.

In relation to the symbolic aspects attributed to wild birds, there were beliefs expressed by the interviewees about the vocalizations of *Turdus rufiventris* and *Euphonia chlorotica* that could stimulate positive attitudes toward them in terms of their maintenance in natural environments, as the former is believed to presage rainfall (a natural event of extreme importance to the human populations in this semiarid region), while the other species is badly thought of as a potential pet (which could protect it to some degree from anthropogenic pressure) [9,98].

## Conclusions

The accumulated bodies of knowledge concerning the avifauna occurring in the semiarid regions of northeastern Brazil and the use of those biological resources in both symbolic and medicinal capacities by human populations demonstrate the cultural importance of this vertebrate group. The results of the present research demonstrated that this accumulated knowledge is an integral part of the cultural heritage of those people and apparently continues to be orally transmitted to current generations.

Some of the bird species cited in the present work are not exclusively used in traditional medicine but have additional symbolic utility as well as food value and use as pets. Additionally, the fact that the interviewees know about the therapeutic effects of birds and in their symbolic values reveals a belief system that is related to cynegetic practices.

It was also observed that birds that retain significant symbolic importance can stimulate either positive or negative attitudes in humans from the perspective of biodiversity conservation. As such, it will be important to undertake specific research focusing on the diverse uses of the avifauna by human populations to determine if the cultural values assigned to them could aid in future programs directed toward biodiversity conservation.

## Competing interests

The authors declare that they have no competing interests.

## Authors’ contributions

DMMB, HFPA, AGCA and RRNA – Writing of the manuscript, literature survey and interpretation; DMMB - Ethnozoological data; DMMB and HFPA – Analysis of taxonomic aspects. All authors read and approved the final manuscript.
